# The Medium-Term Impact of COVID-19 Lockdown on Referrals to Secondary Care Mental Health Services: A Controlled Interrupted Time Series Study

**DOI:** 10.3389/fpsyt.2020.585915

**Published:** 2020-11-26

**Authors:** Shanquan Chen, Rui She, Pei Qin, Anne Kershenbaum, Emilio Fernandez-Egea, Jenny R. Nelder, Chuoxin Ma, Jonathan Lewis, Chaoqun Wang, Rudolf N. Cardinal

**Affiliations:** ^1^Department of Psychiatry, University of Cambridge, Cambridge, United Kingdom; ^2^The Jockey Club School of Public Health and Primary Care, The Chinese University of Hong Kong, Hong Kong, China; ^3^Department of Biostatistics and Epidemiology, Shenzhen University Health Science Center, Shenzhen, China; ^4^Cambridgeshire and Peterborough NHS Foundation Trust, Cambridge, United Kingdom; ^5^Department of Public Health and Primary Care, University of Cambridge, Cambridge, United Kingdom; ^6^College of Public Administration, Central China Normal University, Wuhan, China

**Keywords:** COVID-19/SARS-CoV-2 coronavirus pandemic, lockdown, secondary care mental health services, controlled interrupted time series analysis, comorbidity

## Abstract

To date, there is a paucity of information regarding the effect of COVID-19 or lockdown on mental disorders. We aimed to quantify the medium-term impact of lockdown on referrals to secondary care mental health clinical services. We conducted a controlled interrupted time series study using data from Cambridgeshire and Peterborough NHS Foundation Trust (CPFT), UK (catchment population ~0.86 million). The UK lockdown resulted in an instantaneous drop in mental health referrals but then a longer-term acceleration in the referral rate (by 1.21 referrals per day per day, 95% confidence interval [CI] 0.41–2.02). This acceleration was primarily for urgent or emergency referrals (acceleration 0.96, CI 0.39–1.54), including referrals to liaison psychiatry (0.68, CI 0.35–1.02) and mental health crisis teams (0.61, CI 0.20–1.02). The acceleration was significant for females (0.56, CI 0.04–1.08), males (0.64, CI 0.05–1.22), working-age adults (0.93, CI 0.42–1.43), people of White ethnicity (0.98, CI 0.32–1.65), those living alone (1.26, CI 0.52–2.00), and those who had pre-existing depression (0.78, CI 0.19–1.38), severe mental illness (0.67, CI 0.19–1.15), hypertension/cardiovascular/cerebrovascular disease (0.56, CI 0.24–0.89), personality disorders (0.32, CI 0.12–0.51), asthma/chronic obstructive pulmonary disease (0.28, CI 0.08–0.49), dyslipidemia (0.26, CI 0.04–0.47), anxiety (0.21, CI 0.08–0.34), substance misuse (0.21, CI 0.08–0.34), or reactions to severe stress (0.17, CI 0.01–0.32). No significant post-lockdown acceleration was observed for children/adolescents, older adults, people of ethnic minorities, married/cohabiting people, and those who had previous/pre-existing dementia, diabetes, cancer, eating disorder, a history of self-harm, or intellectual disability. This evidence may help service planning and policy-making, including preparation for any future lockdown in response to outbreaks.

## Introduction

The 2019 novel coronavirus disease (COVID-19) was declared a pandemic by the World Health Organization (WHO) on 11 March 2020 (1). To minimize transmission, “social distancing” or “lockdown” measures were adopted as public health measures globally, including in the United Kingdom (UK), which commenced social distancing from 16 May 2020 and lockdown rules from 23 March 2020.

It is already evident that COVID-19 and lockdown have the potential to lead to pervasive mental health problems. Li et al. (2) surveyed 5,033 individuals from the general population in China following the local onset of COVID-19 and found that 20.4% of people had anxiety or depression or both. Ni et al. (3) surveyed 1,577 adults in China and found that 23.8 and 19.2% of people reported probable anxiety and depression, respectively. Moghanibashi-Mansourieh (4) surveyed 10,754 individuals from the general population in Iran and found that 19.1% of people had severe symptoms of anxiety. A survey in the UK (5) involving 2,198 members of the general population also revealed widespread concerns about COVID-19 and mental health problems. A recent position paper (6) noted that besides the rise in adverse mental symptoms, there is also a risk of increasing numbers of people suffering clinically significant mental disorders. However, the effects of the COVID-19 pandemic and state-imposed lockdown on public mental health in a clinical setting have not been evaluated systematically, and current evidence is mainly based on online surveys. In addition, the evidence relating to long-term consequences of the COVID-19 lockdown upon mental health is sparse (6). Such evidence is urgently needed to inform interventions and for policy makers to formulate an appropriate response.

It has been suggested (7–12) that vulnerable groups such as children and adolescents, pregnant women, older adults, and people with pre-disposing physical and mental health conditions could suffer disproportionately from mental health problems resulting from the current COVID-19 lockdown. Relevant evidence is similarly sparse, and similarly required.

The present study investigated the impact of lockdown on referrals to secondary care mental health clinical services. In addition to examining the overall impact, we also performed a series of sub-group analyses paying special attention to vulnerable groups. As well as the short-term (instantaneous) effect of lockdown, we focused on the subsequent rate of change over time (the longer-term, or medium-term, effect).

## Methods

### Study Design and Participants

We conducted a controlled interrupted time series (CITS) study (13), testing the hypothesis of a causal effect of COVID-19 lockdown, using a historical control group (14). Data were derived from the electronic clinical records of Cambridgeshire and Peterborough NHS Foundation Trust (CPFT), UK. CPFT provides physical and mental health services to a population of approximately 0.86 million. Its records contains patient information recorded during routine treatment, such as age, gender, ethnicity, marital status, referral time, referral destination, diagnoses, and (in free text) some prescription data.

De-identified data were extracted via the CPFT Research Database (NHS Research Ethics 17/EE/0442). We included all records from 2020-01-01 to 2020-03-15 as the pre-lockdown period in 2020, and records from 2020-03-23 to 2020-05-19 as the post-lockdown period. Records from 16 March to 22 March were excluded because this was a transition period (from social distancing to lockdown). Records from 2019-01-01 to 2019-03-15, and from 2019-03-23 to 2019-05-19, were included as the corresponding control year. Weekends were excluded, and bank holidays were excluded from day-by-day analyses.

### Variables

We examined daily referral numbers to mental health service teams. Subgroup CITSs were also conducted, by gender, age, ethnicity, marital status, referral urgency, referral destination (service referred to), and pre-existing diseases. Age was divided into three ranges: 0–19 years (children and adolescents), 20–64 years (working-age adults), and ≥65 years. Ethnicity was categorized into three groups: White, ethnic minorities, and unknown. Marital status was categorized into two groups: cohabiting/married, and single/divorced/widowed. Level of urgency was recorded by clinicians and was grouped into two levels: routine, and urgent/emergency. Referral destination was grouped into 13 team/service categories: crisis teams, liaison psychiatry, personality disorder services, perinatal mental health, autistic spectrum disorder services, eating disorder services, learning disability teams, substance misuse teams, memory or dementia services, psychological therapy services, forensic services, community mental health teams, and others.

Pre-existing diseases were judged based on WHO International Classification of Diseases (ICD-10) codes and on prescription information. Clinician-recorded diagnoses were present in coded data. Medicine information was extracted from free text using GATE-based natural language processing (NLP) software (15). The diseases we focused on included dementia (recorded with ICD-10 codes F00-F03 and G30, or indicated by mentions of cholinesterase inhibitors or glutamate receptor antagonists), substance misuse (F10-F19), severe (serious) mental illness (F20-F29, F30, and F31, or taking antipsychotics), depression (F32 or F33, or taking antidepressants), anxiety (F41 or F42), reaction to severe stress/adjustment disorders (F43), eating disorders (F50), personality disorders (F60-F69), intellectual disability (F70-F79), intentional self-harm (X60-X84), diabetes mellitus (E10-E14, or taking hypoglycemic agents), hypertension or cardiovascular or cerebrovascular disease (I10-I13, I15, I21-I25, and I60-I69, or taking ACE inhibitors, angiotensin-II receptor antagonists, beta blockers, calcium channel antagonists, or diuretics), dyslipidemia (E78, or taking lipid-lowering medications), asthma or chronic obstructive pulmonary disease (COPD) (J44, J45, or taking oral or inhaled corticosteroids, bronchodilators, or anti-inflammatory drugs used for airways disease, accepting that oral corticosteroids may also indicate other inflammatory disorders), and cancer (C00-C97, or taking drugs specifically licensed for cancer). Identification of these diseases were based on CPFT records up to 1 year before a referral was made, except for lifelong diseases including dementia, severe mental illness and the aforementioned physical diseases (for which a record at any time was included). The medicines referred to were selected according to UK National Institute for Health and Care Excellence (NICE) guidelines and are listed in Supplementary Table 1.

### Statistical Analysis

We report referral frequency as mean with standard deviation. For the CITS, data were fitted using the following equation:

(1) $$\begin{array}{r} y\;=\;\beta_{0}\;+\;\beta_{1}\cdot Time\;+\;\beta_{2}\cdot Phase\;+\;\beta_{3}\cdot Phase\cdot Time\\ \;+\;\beta_{4}\cdot Year\;+\;\beta_{5}\cdot Year\cdot Time\;+\;\beta_{6}\cdot Year\cdot Phase\\ \;+\;\beta_{7}\cdot Year\cdot Phase\cdot Time\;+\;\text{Month}\;+\;\text{Weekday}\;+\;\text{error} \end{array}$$

where *y* is the daily number of referrals; *Time* is the time point of data (within a phase); *Phase* indicates “before/after lockdown or equivalent period” (0 before lockdown in 2020 or the equivalent period in 2019, and 1 after lockdown in 2020 or the equivalent period in 2019); *Phase* × *Time* was the time after lockdown in 2020 or the equivalent point in 2019; *Year* indicates 2020 (0 for control data from 2019, or 1 for 2020); *Year* × *Time* was time for 2020 and 0 for 2019; *Year* × *Phase* was 1 after lockdown, or 0 before lockdown and during the control year; *Year* × *Phase* × *Time* was the time after lockdown, or 0 before lockdown and during the control year.

The coefficients in equation 1 are described elsewhere (16) and in Table 2. Two are of particular importance with respect to lockdown. β_6_ reflects the short-term change (instantaneous effect) resulting from lockdown, over and above any equivalent change that may have occurred in 2019. β_7_ was the coefficient of primary interest in this study, and represents the longer-term effect of lockdown, namely the slope change in referral rate following lockdown (after any instantaneous effect and relative to the pre-lockdown slope), over and above any equivalent change in the control year (2019).

Equation 1 was fitted by negative binomial regression, in view of overdispersion seen in the referral numbers. To control for seasonal trends, the day of week and month were also included in the regression. The Breusch–Godfrey and Breusch–Pagan tests were used to check for autocorrelation and heteroskedasticity of residuals, respectively. To interpret the fitted result, a linear approximation to the negative binomial was performed by estimating the marginal effects (ME) and 95% confidence intervals (CI) for the means.

Sensitivity analyses were conducted by examining weekly instead of daily referral numbers.

All statistical analysis were performed using R (version 3.5.0). Statistical significance was defined as *P* < 0.05, and all tests were two-tailed. Missing data were not imputed.

### Patient and Public Involvement

Service user and carer representatives assessed this programme of work and approved it as members of the CPFT Research Database Oversight Committee, but were not involved in the development of this research question or the outcome measures, or in developing plans for the design and implementation of the study.

## Results

**Figure 1:**
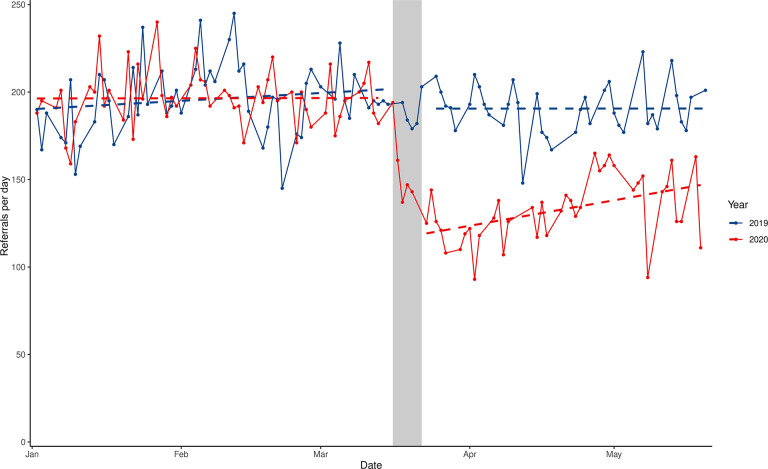
Interrupted time series analysis of referrals to secondary care mental health services in 2020 (red) and 2019 (blue). The dashed line is the model fit. The gray area indicates the transition period from social distancing (16 March 2020) to lockdown (23 March 2020).

Table 1 shows the number of referrals before and after lockdown in 2020, and during the equivalent control periods. After lockdown, there was an immediate decrease in the number of referrals to mental health services. A decrease was also seen in the control period, but the decrease was much larger in 2020 than in 2019. Similar results were observed for subgroups. As shown in Figure 1, there was no difference in the trend between the pre-lockdown period in 2020 and the equivalent period in 2019. However, after lockdown, there was a substantial drop in the referral rate and then an acceleration of referrals compared to the control period.

**Table 1:** Referrals per day to secondary care mental health services, shown as mean (SD).

	**Year 2020**			**Year 2019**		
	**Before lockdown (a)**	**After lockdown (b)**	**Change, b – a**	**Control data for (a)**	**Control data for (b)**	**Change, control (b) – control (a)**
**Overall**	196.48 (15.98)	132.79 (18.87)	−63.69	196 (20.5)	190.55 (14.52)	−5.45
**Gender**						
Female	109.19 (11.7)	73.44 (10.54)	−35.75	108.55 (15.04)	105.47 (10.89)	−3.08
Male	86.62 (8.5)	59.31 (12.16)	−27.31	87.28 (11.8)	84.97 (10.7)	−2.31
**Age**						
0–19	35.23 (8.27)	19 (6.03)	−16.23	32.19 (8.88)	33.39 (8.43)	1.2
20–64	118.31 (12.62)	89.79 (12.42)	−28.52	120.47 (13.44)	114.11 (11.84)	−6.36
≥65	42.94 (7.85)	24 (7.38)	−18.94	43.32 (8.59)	43.03 (6.38)	−0.29
**Ethnicity**						
White	150.73 (15.02)	103.03 (15.01)	−47.7	157.26 (17.67)	152.92 (11.84)	−4.34
Ethnic minorities	12.79 (4.13)	10.26 (3.68)	−2.53	14.74 (3.93)	13.26 (4.94)	−1.48
Unknown	32.96 (5.83)	19.51 (5.53)	−13.45	24 (5.76)	24.37 (4.9)	0.37
**Marital status**						
Single/divorced/widowed	166.44 (14.19)	112.03 (17.75)	−54.41	160.19 (17.66)	156.89 (13.78)	−3.3
Cohabiting/married	30.04 (5.34)	20.77 (5.04)	−9.27	35.81 (7.4)	33.66 (5.26)	−2.15
**Level of urgency**						
Routine	110.5 (14.21)	64.1 (12.59)	−46.4	111.09 (16.49)	106.13 (13.52)	−4.96
Urgent/emergency	85.98 (11.51)	68.69 (14.67)	−17.29	84.91 (12.51)	84.42 (11.72)	−0.49
**Referral destination**						
Crisis teams	77.62 (8.49)	69.95 (10)	−7.67	78.64 (9.34)	73.82 (8.77)	−4.82
Community mental health teams	48.12 (9.21)	22.49 (6.2)	−25.63	44.28 (9.78)	46.13 (8.54)	1.85
Liaison psychiatry	40.21 (7.36)	24.08 (6.87)	−16.13	36.36 (6.93)	35.71 (6.13)	−0.65
Memory/dementia services teams	3.85 (1.95)	2.62 (1.63)	−1.23	7.23 (2.8)	7.45 (3.8)	0.22
Forensic service teams	2.94 (2.02)	2.6 (1.65)	−0.34	6.73 (6.85)	2.92 (1.56)	−3.81
Autistic spectrum disorder teams	4.63 (2)	1.73 (1.08)	−2.9	6.41 (3.35)	6.86 (3.36)	0.45
Eating disorders teams	4.77 (2.24)	3.22 (1.93)	−1.55	4.25 (2.08)	3.81 (1.87)	−0.44
Personality disorder teams	2.85 (2.68)	2.52 (1.91)	−0.33	2.59 (1.57)	3.06 (1.61)	0.47
Learning disability teams	1.98 (0.88)	2 (0.95)	0.02	2.27 (1.36)	2.21 (1.24)	−0.06
Psychological therapy services teams	4.02 (2.2)	2.61 (2.25)	−1.41	1.69 (0.81)	2.22 (1.39)	0.53
Perinatal mental health teams	3.78 (2.06)	3.03 (2.09)	−0.75	3.54 (1.95)	3.78 (1.92)	0.24
Substance misuse teams	5.5 (1.43)	1.88 (1.46)	−3.62	3.8 (2.25)	2.89 (1.45)	−0.91
Other	2.71 (1.86)	1.15 (0.38)	−1.56	3.96 (2.08)	3.59 (1.59)	−0.37
**Pre-existing condition**						
Dementia	12.79 (4.39)	7.85 (3.45)	−4.94	16.74 (5.5)	11.45 (3.42)	−5.29
Substance misuse	5.9 (2.87)	4.61 (2.15)	−1.29	6.49 (3.32)	5.54 (3.17)	−0.95
Serious mental illness	71.31 (9.08)	58.95 (10.75)	−12.36	72.32 (9.98)	64.61 (8.82)	−7.71
Depression	102.04 (11.2)	75.82 (13.06)	−26.22	98.91 (12.51)	90.87 (11.89)	−8.04
Anxiety	7.31 (3.33)	5.72 (2.92)	−1.59	6.98 (2.49)	5.16 (2.72)	−1.82
Eating disorders	3.06 (2.02)	1.87 (0.96)	−1.19	2.92 (1.63)	2.32 (1.39)	−0.6
Reaction to severe stress	10.08 (3.24)	6.97 (3.45)	−3.11	8.02 (3.12)	7.21 (3.73)	−0.81
Personality disorders	15.25 (3.68)	11.69 (3.85)	−3.56	16.75 (4.95)	13.26 (4.32)	−3.49
Intellectual disability	1.33 (0.65)	1.6 (0.89)	0.27	1 (0)	1.75 (0.96)	0.75
Diabetes mellitus	11.96 (4.75)	9.49 (2.76)	−2.47	12.53 (3.89)	12.29 (3.81)	−0.24
Hypertension, cardiovascular, and cerebrovascular disease	40.42 (7.3)	30.79 (6.01)	−9.63	53.92 (8.94)	48.45 (6.93)	−5.47
Cancer	1.3 (0.56)	1.8 (1.01)	0.5	1.39 (0.67)	1.2 (0.41)	−0.19
Dyslipidemia	19.5 (4.36)	16.03 (3.77)	−3.47	28.98 (5.74)	26.26 (5.51)	−2.72
Asthma/COPD	17.08 (4.55)	12.21 (4.2)	−4.87	21.7 (4.88)	18.76 (4.95)	−2.94
Intentional self-harm	5.76 (2.52)	5.26 (2.33)	−0.5	5.73 (2.49)	5.08 (2.8)	−0.65

Table 2 shows the overall regression results. After controlling for seasonal trends, neither the mean referral rate (β_4_, 6.06, 95% CI −6.41 to 18.53) nor the time trend in referral rate (β_5_, −0.21, CI −0.58 to 0.16) was significantly different between the pre-lockdown period in 2020 and its control period in 2019. This indicated that 2019 could be treated as a homogenous counterpart to 2020, before lockdown. Controlling for other temporal effects, lockdown resulted in an immediate decrease in the total number of referrals, by 72 referrals·day^−1^ (short-term effect, β_6_, CI −85.13 to −58.14), and a subsequent acceleration in referrals, of 1.21 referrals·day^−2^ (medium-term effect, β_7_, CI 0.41 to 2.02). There was no significant autocorrelation or heteroskedasticity in the fitted residuals (see Supplementary Table 2, Supplementary Figure 1).

**Table 2:** Overall controlled interrupted time-series analysis: negative binomial regression adjusted for seasonal effects (month and day of week). The dependent variable is the referral rate (referrals·day^−1^).

**Variable**	**Coefficient**	**Explanation**	**Units**	**Marginal effect (95% CI)**	***P***
Time	β_1_	Trend in 2019 before 16 March	Referrals·day^−2^	0.29 (−0.19, 0.77)	0.2365
Phase	β_2_	Step change in the referral rate in 2019, from before 16 March to after 22 March	Referrals·day^−1^	**−10.54 (−20.87**, **−0.21)**	**0.0455**
Phase × Time	β_3_	Trend change (rate of change of referral rate) in 2019 after 22 March	referrals·day^−2^	−0.18 (−0.58, 0.21)	0.3609
Year	β_4_	The difference in the referral rate between the pre-lockdown period in 2020 and the equivalent control period in 2019	Referrals·day^−1^	6.06 (−6.41, 18.53)	0.3411
Year × Time	β_5_	The difference in the rate of change (in referral rate) over time between the pre-lockdown period in 2020 and the equivalent control period in 2019	Referrals·day^−2^	−0.21 (−0.58, 0.16)	0.2737
Year × Phase	β_6_	Short-term (instantaneous) effect of lockdown. The difference between the referral rate during the post-lockdown period in 2020 and that during the equivalent control period in 2019, each relative to the referral rate during the pre-lockdown period	Referrals·day^−1^	**−71.64 (−85.13**, **−58.14)**	**<0.0001**
Year × Phase × Time	β_7_	Longer-term (medium-term) effect of lockdown. Rate of change (in referral rate) after lockdown, over and above any corresponding change during the equivalent control period	Referrals·day^−2^	**1.21 (0.41, 2.02)**	**0.0032**

*Bold text indicates p < 0.05*.

**Figure 2:**
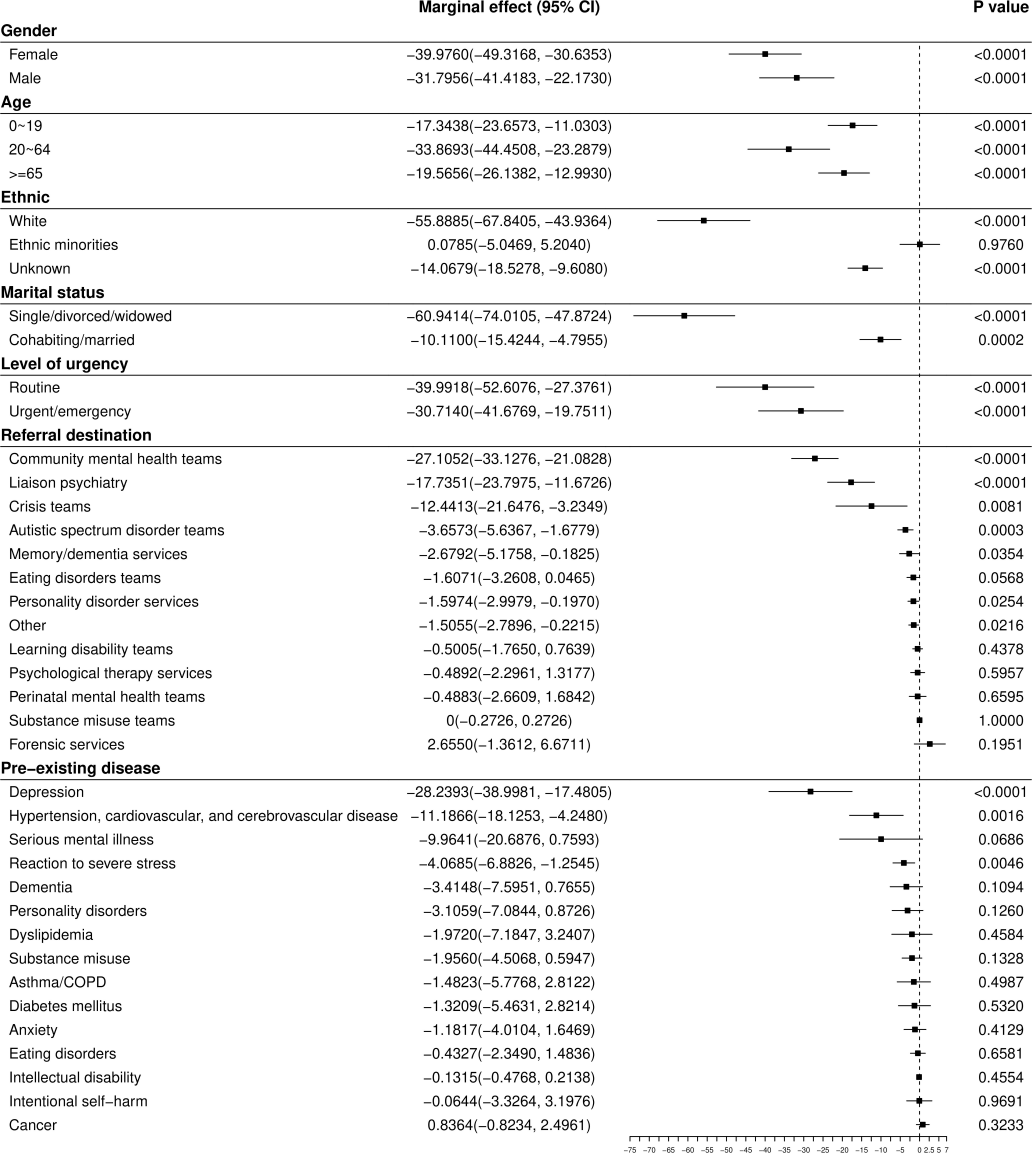
Short-term (β_6_) effects of COVID-19 lockdown on the daily number of referrals to mental health services for subgroups. Units are referrals·day^−1^.

**Figure 3:**
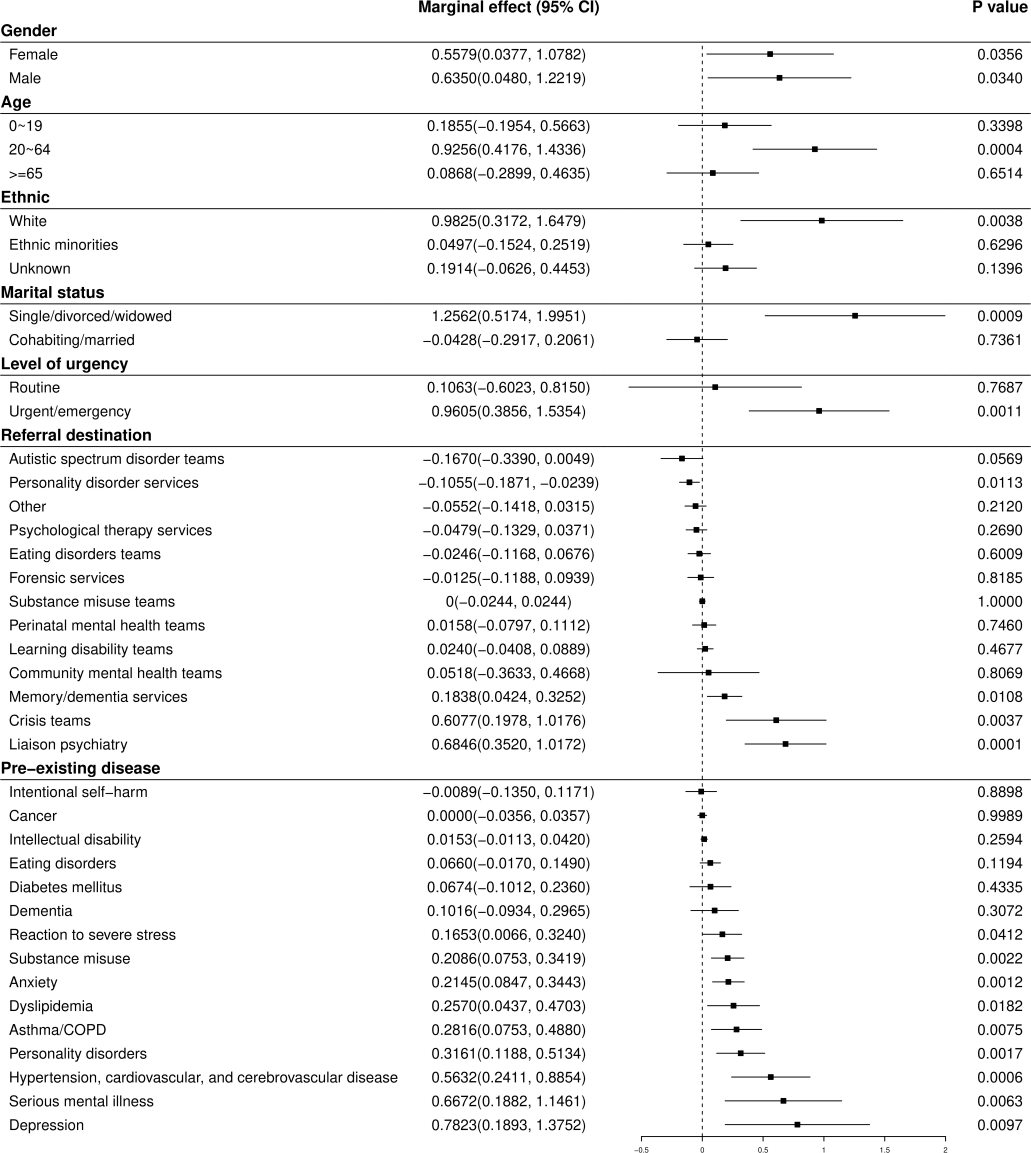
Medium-term (β_7_) effects of COVID-19 lockdown on the time trend in referrals to mental health services for subgroups. Units are referrals·day^−2^.

Subgroup analyses are shown in Figure 2 (short-term effect) and Figure 3 (longer-term effect). The immediate decrease in referral rate to mental health services (β_6_) remained statistically significant for both genders; all age groups; those of White ethnicity; both levels of marital status; both levels of urgency; referrals to community mental health teams, liaison psychiatry, crisis teams, autistic spectrum disorder teams, memory/dementia services, and personality disorder services; and for those who had pre-existing depression, hypertension/cardiovascular/cerebrovascular disease, and reaction to severe stress. After these immediate effects of lockdown, there was an acceleration (β_7_) in urgent/emergency referrals (0.96 referrals·day^−2^, CI 0.39 to 1.54), and in referrals to liaison psychiatry (0.68, CI 0.35 to 1.02) and mental health crisis teams (0.61, CI 0.20 to 1.02). The acceleration remained significant for both genders (females 0.56, CI 0.04 to 1.08, males 0.64, CI 0.05 to 1.22); working-age adults (0.92, CI 0.42 to 1.43); those of White ethnicity (0.98, CI 0.32 to 1.65), those living alone (1.26, CI 0.52 to 2.00); and those who had previous or pre-existing depression (0.78, CI 0.19 to 1.34), severe mental illness (0.67, CI 0.19 to 1.15), hypertension/cardiovascular/cerebrovascular disease (0.56, CI 0.24 to 0.89), personality disorders (0.32, CI 0.12 to 0.51), asthma/COPD (0.28, CI 0.08 to 0.49), dyslipidemia (0.26, CI 0.04 to 0.47), anxiety (0.21, CI 0.08 to 0.34), substance misuse (0.20, CI 0.06 to 0.34), or reactions to severe stress (0.17, CI 0.01 to 0.32). No significant longer-term rate changes were observed for children and adolescents, older adults, people of ethnic minorities, married/cohabiting people, or those who had pre-existing dementia, diabetes, cancer, eating disorder, a history of intentional self-harm, or intellectual disability.

Sensitivity analyses confirmed this pattern of results (Supplementary Figures 2, 3).

## Discussion

To our knowledge, this is the first study to evaluate systematically the longer-term effects of COVID-19 lockdown on a population's mental health in a clinical setting via a detailed examination of the trajectory of referral numbers. With a rigorous study design to detect causal effects in time series data, we found that after an initial decrease (17), lockdown led in the medium term to an acceleration in the rate of referrals. The significant longer-term effects revealed in our study are generally consistent with the mental health concerns associated with COVID-19 raised by others. However, the finding was to some extent inconsistent with a study conducted by Wang et al. (18). They conducted two online cross-sectional studies in China, 4 weeks apart, and found no significant changes in scores of depression, anxiety, and stress. This might be partially attributed to the variations in methodology and characteristics of study participants (e.g., the online design, the relatively small sample size of Wang's study, and the measurement of self-reported mental health problems). Given that our study was conducted in a clinical setting, had a longer duration, and used a more rigorous design for causal inference, we suggest our findings are more representative of clinical practice compared with previous cross-sectional or online surveys that used screening scales for mental disorders.

The significant medium-term effects of COVID-19 lockdown on referrals to mental health services were observed in multiple subgroups, supporting the validity of the findings. For instance, after lockdown, referrals accelerated again across genders. Unexpectedly, no such acceleration was observed among those aged ≤19 or ≥65, who have been suspected to be especially vulnerable to mental health problems resulting from social distancing or lockdown (10, 12, 19–21), even though all age groups exhibited the significant instantaneous drop in referrals at lockdown. The lack of acceleration in referrals for older adults after lockdown was also to some extent inconsistent with a study (22) conducted in Hong Kong at the time of the severe acute respiratory syndrome (SARS) epidemic in 2003, which found SARS to be associated with a 30% increase in suicide in those aged ≥65. A possible reason why referrals did not accelerate again for this group may have been that some older people who lived alone and without close support may have lost their capability to access services as usual (19). Fear of contracting COVID-19 infection in a healthcare setting might also have deterred people from seeking help for their mental health, especially for the elderly (for whom a UK public campaign emphasized their additional vulnerability).

Similarly, we did not observe a significant post-lockdown acceleration in referrals for those from ethnic minorities, though this acceleration was significant for those of White ethnicity (indeed, the acceleration for the ethnic minority group was significantly less). Although the immediate lockdown-associated change in referrals for those of ethnic minorities was also not significant, this observation, coupled with the fact that ethnic minority groups are already at elevated risk of dying from COVID-19 in the UK (23), raises the concern that they may not be obtaining mental health support proportionally following lockdown. Together with the lack of acceleration observed in children/adolescents and elderly people, these findings should prompt consideration as to whether these represent disadvantaged groups requiring active mental health intervention.

A significant contribution of this study is that we explored the post-lockdown trends in referral by service referred to, which indirectly suggests the type and degree of mental disorders to which the lockdown may lead. The post-lockdown acceleration in referrals to liaison psychiatry and crisis teams is in keeping with the fact that the acceleration was for urgent/emergency rather than routine referrals (which may also reflect a reduction in routine services). It has been hypothesized (6) and suggested by two cross-sectional studies that COVID-19 might lead to an increase in depressive symptoms and anxiety amongst pregnant women (24, 25). In contrast, our study found neither an initial change nor a subsequent rate of change in the referral rate to perinatal mental health teams. This may reflect service supply as well as patient demand factors, but this inconsistency clearly shows that the increase of mental symptoms assessed by screening scales may not necessarily predict clinical referrals for mental disorders.

This study also provides evidence about the longer-term effect of lockdown on referrals for patients with pre-existing diseases, suggesting indirectly which groups of patients suffer more mental ill health during the lockdown, and providing implications for resource planning and clinical management. Our results give support to concerns that those with severe mental illness (11, 26, 27) would be particularly vulnerable to the long-term effects of lockdown. Our results also indicate that patients with previous/pre-existing depression, personality disorders, anxiety, substance misuse, and reactions to severe stress also required accelerating rates of referral after lockdown, and may need early intervention to prevent the relapse or aggravation of pre-existing mental disorders or the emergence of new problems. Previous cross-sectional studies (28–31) indicated that people with poorer self-reported health status, or comorbid chronic diseases, were more likely to report mental health problems. Our study found that patients who had hypertension, cardiovascular, cerebrovascular disease, asthma, COPD, or dyslipidemia showed accelerating rates of mental health referrals after COVID-19 lockdown. [Hypertension and dyslipidemia are perhaps notable as largely asymptomatic disorders; the association here may reflect an indirect relationship via other conditions, be they physical, such as other cardiometabolic diseases, or psychiatric, such as depression (32).] Special attention may need to be paid to the mental health of patients with chronic cardiovascular or respiratory diseases.

Notably, referral types that showed the greatest initial drop (Figure 2) tended to show the largest subsequent rate increase (Figure 3). Referral numbers reflect the actions of professionals, and the instantaneous drop reflected, in part, attempts by health services to minimize the probability of patients entering high-risk areas for infection unnecessarily (17), and to release resources for the management of patients with COVID-19. However, it is less clear that this explains the subsequent increase in referral rates. Therefore, although it is possible that the longer-term effects identified in this study may not result solely from the social consequences of COVID-19 lockdown but also from adaptation and “rebound” of the health system from the initial sharp decline resulting from lockdown, the prior probability that the changes observed were caused by the pandemic is extremely high and this may have been direct or via a range of indirect mechanisms. For example, these effects may reflect both changes in supply (reduced provision of health services, and the prioritization of urgent over routine referrals with discouragement of routine referral) as well as changes in patient-led demand (17). Regression to the mean (“what went down most, came up most”) cannot explain our data fully. Routine referrals, for example, decreased substantially but then did not increase accordingly. This may have reflected service-driven pressures to reduce non-urgent activity. Referrals for people who were married/cohabiting dropped at lockdown, but then did not increase subsequently, perhaps reflecting less mental health need in this group compared with people who live alone. Referrals for people with pre-existing asthma/COPD did not decrease significantly at lockdown but still increased subsequently. This is in keeping with the suggestion that COVID-19 imposes particular additional mental stress on patients with pre-existing respiratory disease (33, 34).

Our findings are, of course, correlational and do not prove causality. However, a number of potential mitigating strategies may be applicable to “lockdown.” First, mental health services must to be able to deliver services to all in need, by operating a flexible mix of in-person and remote (e.g., videoconferencing) consultations to balance clinical and infection control requirements; there is not a “one size fits all” approach (35). Secondly, referrers should be encouraged to have a low threshold for potentially vulnerable populations (including children, the elderly, those of ethnic minorities, those who are socially isolated, and those with pre-existing mental disorders and physical comorbidities). Thirdly, evidence-based measures to support population mental health (36)—including the promotion of community cohesion (37)—are likely to be helpful, and to require government support (36). Such interventions may be constrained additionally by the economic effects of the pandemic, and governments must balance economic and health considerations. However, it is notable that personal economic loss during lockdown is associated with worsening of mental health, particularly an increase in depressive symptoms (38), and that explicit consideration of the trade-offs between infective and economic/wellbeing considerations may be required for maximum public benefit (39).

Our study had several limitations. First, we used clinical data up to ~2 months after UK lockdown; this may not be long enough for some effects of lockdown to have become evident. Second, the referral data used in this study could only reflect problems severe enough to require referral to secondary care mental health services. Thus, this study may underestimate longer-term effects, as some individuals may have been suffering mental health problems resulting from COVID-19/lockdown but used primary care services only, or did not seek mental health services at all. The present findings thus highlight mental health needs and a disease burden that should be prioritized for attention and treatment. Third, classifying by diagnosis instead of referral service would be more helpful to establish disease patterns, but frequent missing coded data on diagnosis following urgent or emergency referrals impeded our analysis. Fourth, some types of referral, for example for people with diabetes or cancer, were relatively rare in the dataset, posing a challenge (via low power) to identifying the effects of lockdown upon them. We addressed this by performing sensitivity analyses examining weekly instead of daily referral numbers; this confirmatory analysis supported our primary results. Finally, our findings may not generalize to other areas, such as with very different COVID-19 infection rates, or health service organization. However, measures such as social distancing and lockdown are being adopted in similar ways internationally, so the results of the present study may be useful for other regions and countries.

## Conclusions

The present study, using clinical records and the optimal causal inference study design for time series analysis, is compatible with the hypothesized negative long-term impact of lockdown on mental health. After an initial drop in referrals, we observed a post-lockdown acceleration in urgent and emergency mental health referrals. This acceleration was significant for adults of working age, people who live alone, and those with a broad range of pre-existing mental disorders and a subset of physical comorbidities. No such acceleration was observed for children/adolescents, older adults, and those from ethnic minorities; one possibility is that they may be facing insufficient access to mental health services. The timely evidence in this study could help the response of mental health systems and policy to support population mental health, as well as in preparation for any future lockdown in response to further outbreaks.

## Data Availability Statement

The data analyzed in this study is subject to the following licenses/restrictions: Patient-level data is not publicly available, under NHS Research Ethics terms. Source code and summary data are available on request. Requests to access these datasets should be directed to Rudolf N. Cardinal, rnc1001@cam.ac.uk.

## Ethics Statement

The studies involving human participants were reviewed and approved by the NHS Health Research Authority Cambridge Central Research Ethics Committee (reference 17/EE/0442) and CPFT Research Database Oversight Committee. Written informed consent from the participants' legal guardian/next of kin was not required to participate in this study in accordance with national legislation and institutional requirements.

## Author Contributions

SC and RC contributed to the study design and wrote the first draft. SC and JL processed the data. SC conducted the statistical analyses. All authors edited and approved the final manuscript.

## Conflict of Interest

RC consults for Campden Instruments Ltd and receives royalties from Cambridge University Press, Cambridge Enterprise, and Routledge. The remaining authors declare that the research was conducted in the absence of any commercial or financial relationships that could be construed as a potential conflict of interest.
