# Supplementary material for: The Medium-Term Impact of COVID-19 Lockdown on Referrals to Secondary Care Mental Health Services: A Controlled Interrupted Time Series Study
Source: Front Psychiatry. 2020 Nov 26;11:585915. doi: 10.3389/fpsyt.2020.585915 (PMC7726266; doi:10.3389/fpsyt.2020.585915)

frontiers

Supplementary Material

The medium-term impact of COVID-19 lockdown on referrals to secondary care mental health services: A controlled interrupted time series study (Chen et al.)

Supplementary Table 1. List of medicines.

Cholinesterase inhibitors and glutamate receptor antagonists	**Cholinesterase inhibitors:**<br>Donepezil, galantamine, rivastigmine<br>**Glutamate receptor antagonists:**<br>Memantine
Antipsychotics	**First-generation:**<br>Benperidol, chlorpromazine, flupentixol, fluphenazine, haloperidol, levomepromazine, pericyazine, perphenazine, pimozide, pipotiazine, prochlorperazine, promazine, trifluoperazine, zuclopenthixol<br>**Second-generation:**<br>Asenapine, amisulpride, aripiprazole, iloperidone, lurasidone, olanzapine, paliperidone, quetiapine, risperidone, sertindole, sulpiride, ziprasidone, zotepine
Antidepressants	Agomelatine, amitriptyline, bupropion, citalopram, clomipramine, dosulepin, doxepin, duloxetine, escitalopram, fluoxetine, fluvoxamine, imipramine, isocarboxazid, lofepramine, maprotiline, mianserin, mirtazapine, moclobemide, nefazodone, nortriptyline, paroxetine, phenelzine, reboxetine, sertraline, tranylcypromine, trazodone, trimipramine, tryptophan, venlafaxine, vortioxetine
Hypoglycaemic agents	Acarbose, alogliptin, canagliflozin, dapagliflozin, empagliflozin, exenatide, glibenclamide, gliclazide, glimepiride, glipizide, insulin, linagliptin, liraglutide, lixisenatide, metformin, nateglinide, pioglitazone, repaglinide, saxagliptin, sitagliptin, tolbutamide, vildagliptin
ACE inhibitors	Captopril, enalapril, fosinopril, imidapril, lisinopril, perindopril, quinapril, ramipril, trandolapril
Angiotensin-II receptor antagonists	Azilsartan, candesartan, eprosartan, irbesartan, losartan, olmesartan, telmisartan, valsartan
Beta blockers	Acebutolol, atenolol, bisoprolol, carvedilol, celiprolol hydrochloride<br>Labetalol, metoprolol, nadolol, nebivolol, pindolol, sotalol
Calcium channel antagonists	Amlodipine, diltiazem, elodipine, felodipine, lacidipine, lercanidipine, nicardipine, nifedipine, nimodipine, verapamil
Diuretics	**Thiazides:**<br>Bendroflumethiazide, chlortalidone, indapamide, metolazone, xipamide<br>**Loop diuretics:**<br>Bumetanide, furosemide, torasemide<br>**Potassium-sparing diuretics:**<br>Amiloride, triamterene<br>**Aldosterone antagonists:**<br>Eplerenone, spironolactone
Lipid-lowering medication	**Statins:**<br>Atorvastatin, fluvastatin, pravastatin, rosuvastatin, simvastatin<br>**Others:**<br>Colestyramine, colestipol, ezetimibe, fenofibrate
Corticosteroids, bronchodilators and other anti-inflammatory drugs for airways disease	**Corticosteroids:**<br>Alclometasone, betamethasone, clobetasol, clobetasone butyrate, fluocortolone, dexamethasone, fludrocortisone, fludroxycortide, flumetasone, fluocinolone, fluocinonide, fluorometholone, hydrocortisone, loteprednol, methylprednisolone, mometasone, prednisolone, triamcinolone<br>**Inhaled corticosteroids:**<br>Beclomethasone, budesonide, ciclesonide, fluticasone<br>**Bronchodilators and anti-inflammatory drugs used for airways disease:**<br>Aminophylline, formoterol, ipratropium, mepolizumab, montelukast, nedocromil sodium, omalizumab, reslizumab, salbutamol, salmeterol, sodium cromoglicate, terbutaline, theophylline, tiotropium
Drugs implying cancer	Docetaxel, clodronate, raloxifene, anastrozole, paclitaxel, pertuzumab, tamoxifen, trastuzumab, abiraterone acetate, bicalutamide, denosumab, enzalutamide

Supplementary Table 2. Results of the Breusch–Godfrey and Breusch–Pagan tests.

**Breusch–Godfrey test for serial correlation of order up to 16**
LM test = 22·81, *df* = 16, *p* = 0·119
**Studentized Breusch–Pagan test**
BP = 18·07, *df* = 12, *p* = 0·114

Supplementary Figure 1. Residuals from negative binomial regression.

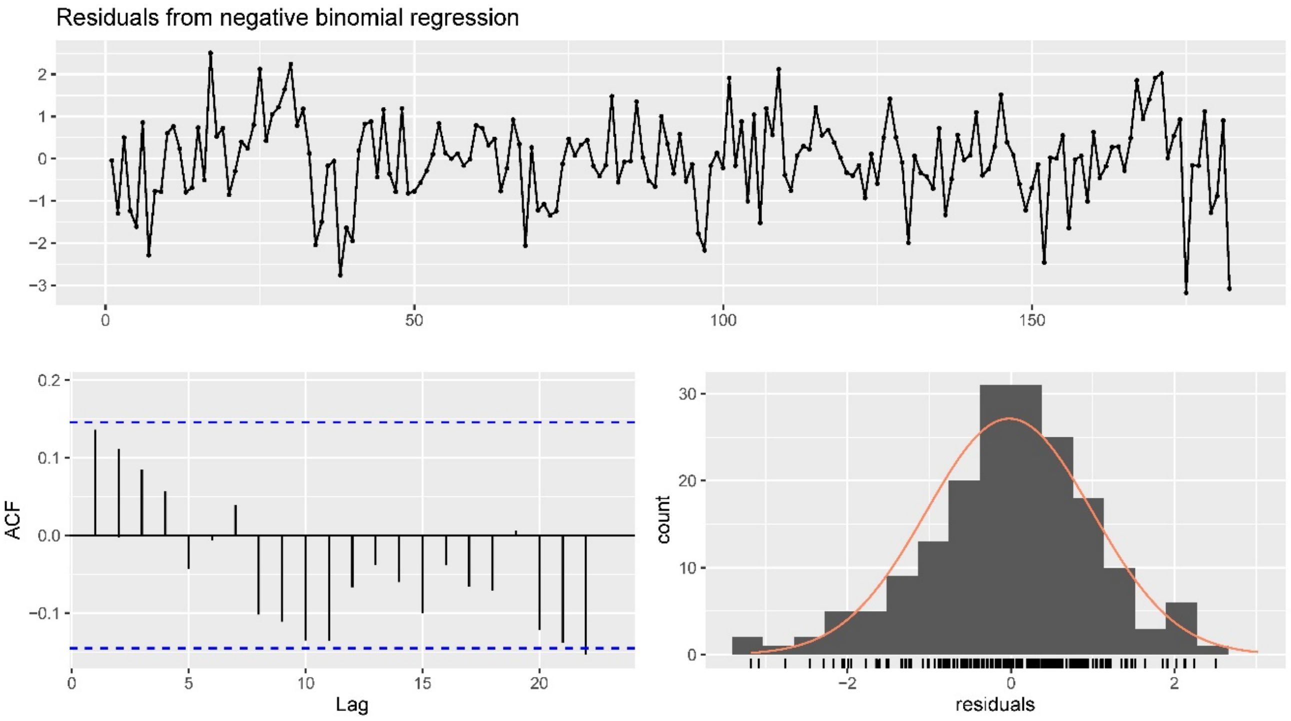

Supplementary Figure 2. Short-term (β_6) effects of COVID-19 lockdown on the referral rate to mental health services, for subgroups (sensitivity analysis by week). Units are referrals·week^{-1}.

	Marginal effect (95% CI)	P value
**Gender**		
Female	−227.7797(−303.1756, −152.3839)	<0.0001
Male	−181.9480(−244.9054, −118.9905)	<0.0001
**Age**		
0~19	−104.2420(−146.7998, −61.6843)	<0.0001
20~64	−215.2192(−277.9167, −152.5217)	<0.0001
>=65	−95.7343(−140.7995, −50.6690)	<0.0001
**Ethnic**		
White	−321.7023(−415.1450, −228.2596)	<0.0001
Unknown	−77.7836(−105.2088, −50.3584)	<0.0001
Ethnic minorities	−12.1018(−37.2527, 13.0492)	0.3456
**Marital status**		
Single/divorced/widowed	−373.2117(−480.4314, −265.9920)	<0.0001
Cohabiting/married	−40.7193(−64.9953, −16.4433)	0.0010
**Level of urgency**		
Routine	−228.0709(−321.7292, −134.4127)	<0.0001
Urgent/emergency	−182.2337(−237.7803, −126.6872)	<0.0001
**Referral destination**		
Community mental health teams	−137.1617(−189.5072, −84.8162)	<0.0001
Liaison psychiatry	−130.7174(−161.9621, −99.4727)	<0.0001
Crisis teams	−78.7932(−128.7576, −28.8288)	0.0020
Memory/dementia services	−25.2865(−33.0971, −17.4759)	<0.0001
Perinatal mental health teams	−9.2144(−16.6057, −1.8230)	0.0145
Autistic spectrum disorder teams	−6.9462(−19.2863, 5.3938)	0.2699
Other	−6.5013(−12.7970, −0.2056)	0.0430
Psychological therapy services	−6.2100(−15.9612, 3.5412)	0.2120
Learning disability teams	−5.5120(−9.3059, −1.7182)	0.0044
Eating disorders teams	−4.2162(−13.7426, 5.3103)	0.3857
Substance misuse teams	−0.5583(−5.5239, 4.4073)	0.8256
Personality disorder services	6.2535(−8.0127, 20.5196)	0.3903
Forensic services	8.6485(−12.3236, 29.6206)	0.4189
**Pre−existing disease**		
Depression	−183.8441(−240.3895, −127.2987)	<0.0001
Serious mental illness	−111.2639(−155.2345, −67.2933)	<0.0001
Hypertension, cardiovascular, and cerebrovascular disease	−85.9212(−121.6497, −50.1926)	<0.0001
Asthma/COPD	−49.8885(−63.9540, −35.8231)	<0.0001
Dyslipidemia	−34.8573(−59.3441, −10.3706)	0.0053
Personality disorders	−31.8882(−49.7924, −13.9840)	0.0005
Substance misuse	−24.3804(−33.8365, −14.9243)	<0.0001
Reaction to severe stress	−22.5913(−38.4125, −6.7700)	0.0051
Diabetes mellitus	−21.8894(−38.8541, −4.9247)	0.0114
Anxiety	−17.5476(−31.7382, −3.3569)	0.0154
Dementia	−11.5463(−37.9099, 14.8173)	0.3907
Eating disorders	−9.9966(−13.9179, −6.0753)	<0.0001
Intellectual disability	−1.1803(−2.5863, 0.2258)	0.0999
Cancer	2.4370(−3.7508, 8.6247)	0.4402
Intentional self−harm	3.9296(−11.4439, 19.3031)	0.6164

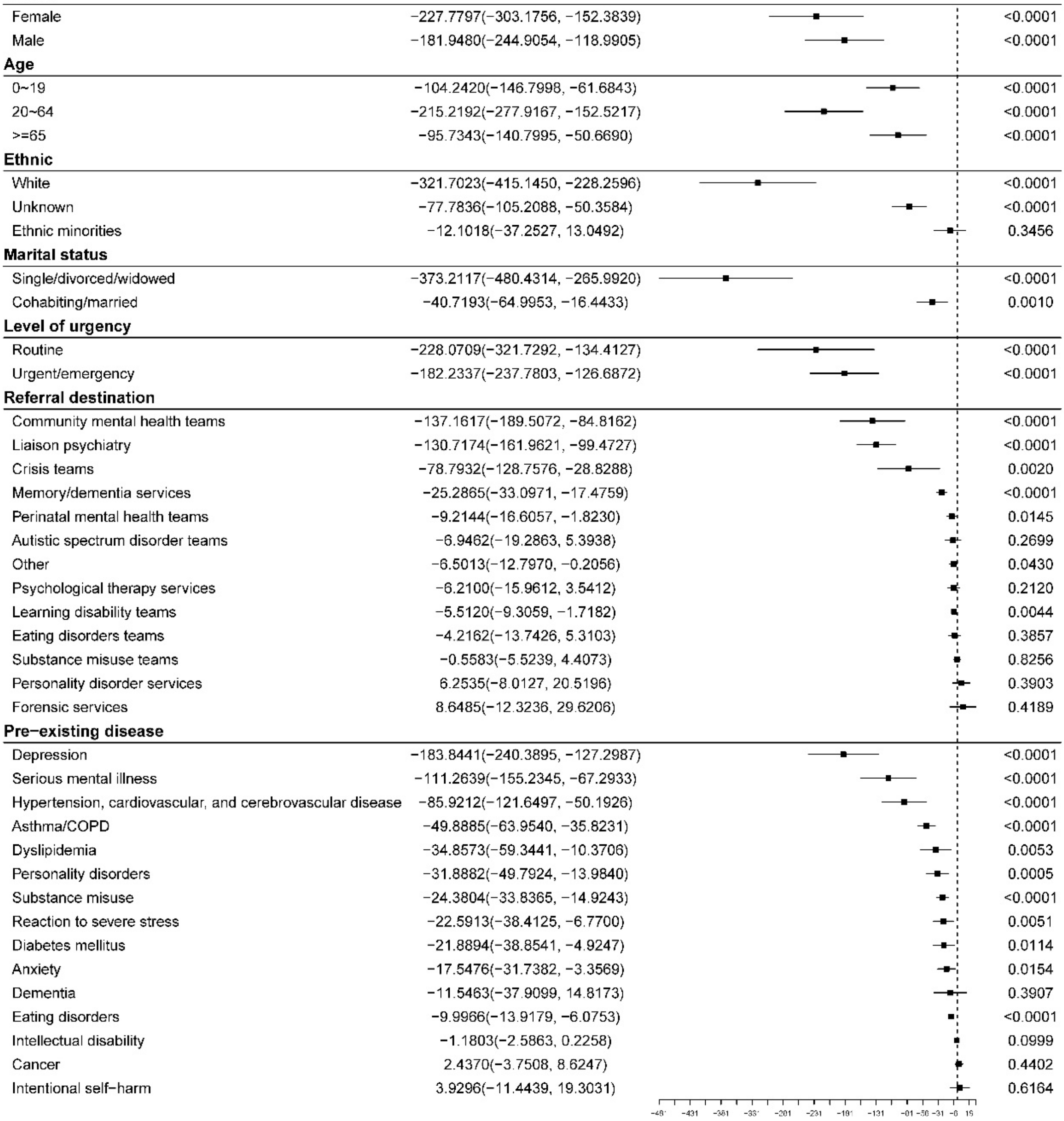

Supplementary Figure 3. Medium-term (β_7) effects of COVID-19 lockdown on the time trend in the referral rate to mental health services, for subgroups (sensitivity analysis by week). Units are referrals·week^{-2}.

	Marginal effect (95% CI)	P value
**Gender**		
Female	20.2270(1.4372, 39.0168)	0.0349
Male	17.4124(0.7070, 34.1177)	0.0411
**Age**		
0~19	7.9708(−3.9880, 19.9296)	0.1914
20~64	26.7102(9.2988, 44.1215)	0.0026
>=65	3.7259(−6.5865, 14.0384)	0.4788
**Ethnic**		
White	29.6759(5.3512, 54.0007)	0.0168
Ethnic minorities	1.3538(−3.8176, 6.5251)	0.6079
Unknown	7.5907(0.7422, 14.4391)	0.0298
**Marital status**		
Single/divorced/widowed	37.0764(10.9783, 63.1746)	0.0054
Cohabiting/married	1.3582(−5.8015, 8.5180)	0.7100
**Level of urgency**		
Routine	10.2942(−10.7868, 31.3752)	0.3385
Urgent/emergency	25.1293(9.5666, 40.6920)	0.0016
**Referral destination**		
Autistic spectrum disorder teams	−5.0185(−8.5908, −1.4462)	0.0059
Personality disorder services	−2.1197(−4.4756, 0.2362)	0.0778
Other	−1.1142(−3.3669, 1.1386)	0.3324
Psychological therapy services	−0.8411(−2.8941, 1.2120)	0.4220
Substance misuse teams	−0.5648(−1.6409, 0.5113)	0.3036
Eating disorders teams	−0.5645(−2.9350, 1.8060)	0.6407
Forensic services	0.5256(−2.8510, 3.9022)	0.7603
Learning disability teams	0.8027(−0.6972, 2.3027)	0.2942
Perinatal mental health teams	1.3863(−0.7933, 3.5659)	0.2125
Memory/dementia services	4.6571(1.8799, 7.4344)	0.0010
Community mental health teams	5.4970(−7.8362, 18.8302)	0.4191
Crisis teams	14.5251(2.3162, 26.7340)	0.0197
Liaison psychiatry	17.1753(9.1371, 25.2135)	<0.0001
**Pre−existing disease**		
Intentional self−harm	−0.4286(−3.5888, 2.7315)	0.7903
Cancer	−0.1218(−1.0844, 0.8408)	0.8042
Intellectual disability	0.2639(−0.3066, 0.8344)	0.3646
Dementia	2.2386(−2.0634, 6.5405)	0.3078
Eating disorders	2.2735(0.3972, 4.1497)	0.0176
Diabetes mellitus	2.4259(−1.7234, 6.5751)	0.2518
Reaction to severe stress	3.6904(−1.0754, 8.4561)	0.1291
Anxiety	4.9929(1.6020, 8.3838)	0.0039
Substance misuse	6.1771(2.6298, 9.7245)	0.0006
Dyslipidemia	6.5478(−0.0587, 13.1543)	0.0521
Personality disorders	8.0096(3.0284, 12.9908)	0.0016
Asthma/COPD	9.4478(4.3760, 14.5197)	0.0003
Hypertension, cardiovascular, and cerebrovascular disease	15.0245(5.5172, 24.5318)	0.0020
Serious mental illness	20.9799(8.3103, 33.6496)	0.0012
Depression	24.7839(9.0152, 40.5526)	0.0021

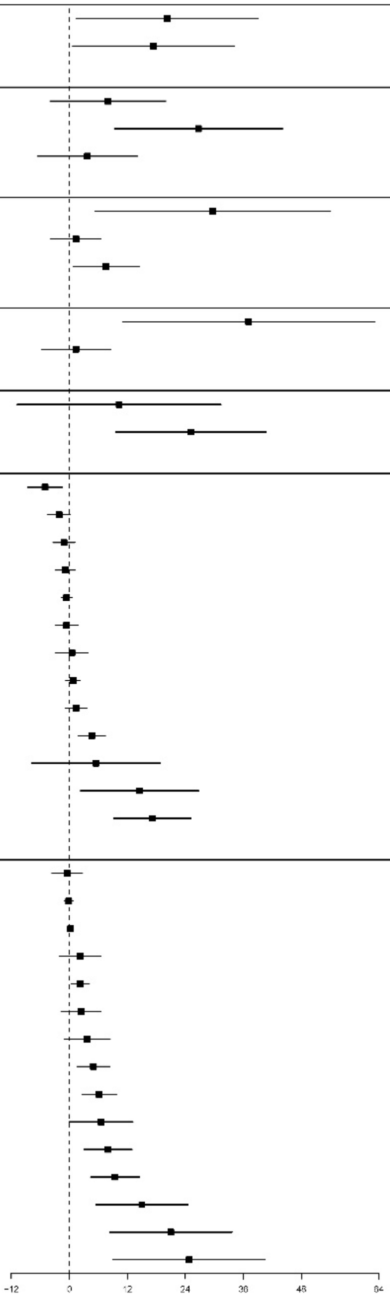

Supplement: Supplementary file 1 [file Data_Sheet_1.PDF]
